# Synthesis of Short-Chain Alkyl Butyrate through Esterification Reaction Using Immobilized *Rhodococcus* Cutinase and Analysis of Substrate Specificity through Molecular Docking

**DOI:** 10.4014/jmb.2211.11022

**Published:** 2022-12-02

**Authors:** Seok-Jae Won, Joung Han Yim, Hyung Kwoun Kim

**Affiliations:** 1 Department of Biotechnology, The Catholic University of Korea, Bucheon 14662, Republic of Korea; 2 Korea Polar Research Institute, Incheon 406-840, Republic of Korea

**Keywords:** Cutinase, esterification, alkyl butyrate, molecular docking, substrate specificity

## Abstract

Alkyl butyrate with fruity flavor is known as an important additive in the food industry. We synthesized various alkyl butyrates from various fatty alcohol and butyric acid using immobilized *Rhodococcus* cutinase (*R*cut). Esterification reaction was performed in a non-aqueous system including heptane, isooctane, hexane, and cyclohexane. As a result of performing the alkyl butyrate synthesis reaction using alcohols of various chain lengths, it was found that the preference for the alcohol substrate had the following order: C6 > C4 > C8 > C10 > C2. Through molecular docking analysis, it was found that the greater the hydrophobicity of alcohol, the higher the accessibility to the active site of the enzyme. However, since the number of torsions increased as the chain length increased, it became difficult for the hydroxyl oxygen of the alcohol to access the ^γ^O of serine at the enzyme active site. These molecular docking results were consistent with substrate preference results of the *R*cut enzyme. The *R*cut maintained the synthesis efficiency at least for 5 days in isooctane solvent. We synthesized as much as 452 mM butyl butyrate by adding 100 mM substrate daily for 5 days and performing the reaction. These results show that *R*cut is an efficient enzyme for producing alkyl butyrate used in the food industry.

## Introduction

Cutinase (E.C. 3.1.1.74), a serine hydrolase belonging to the α/β hydrolytic superfamily, has a catalytic triad of Ser-His-Asp in its active site [1]. Cutinase can decompose esters, polyesters, and triacylglycerol in an aqueous system. It can perform reverse reactions such as esterification and transesterification in a non-aqueous system [2,3]. These characteristics are similar to those of lipase or esterase. Although many experiments related to the synthesis of various ester compounds using lipase or esterase have been performed, cutinase has been used relatively rarely. This is because there are relatively few wild strains that produce cutinase, and the disulfide bond present in cutinase makes it difficult for it to be expressed in *Escherichia coli* cells [4-6]. In the case of substrate specificity of lipase, it shows a high affinity for carbon chains longer than C8. Esterases have a high affinity for very short carbon chains such as C1 or C2 [5]. Cutinase is known to have a high affinity for C4 to C10 carbon chains. In the ester synthesis reaction using *Thermobifida fusca* cutinase, this enzyme shows a high activity for C3 to C8 carbon chains [7]. *Burkholderia cepacia* NRRL B 2320 cutinase shows a high activity for C4 and C5 carbon chains [8]. As such, it might be efficient to synthesize short chain esters using cutinases.

Short aliphatic esters refer to ester compounds of acids and alcohols with less than 10 carbon chains [9]. They are naturally present in fruits and have a refreshing scent [10]. Thus, they are widely used as important additives in various industries such as food, cosmetics, and pharmaceutical industries [11, 12]. For example, ethyl butyrate is naturally present in pineapples, mangoes, and bananas. It has been applied as an additive in the food industry [13]. Butyl butyrate can be extracted from certain naturally occurring plants. It can also be produced by fermentation. Due to its pineapple flavor, it is widely used as an additive in the food industry [14]. Hexyl butyrate is used as an ingredient to flavor fruit, beer, and citrus. Octyl butyrate is used to flavor peach and strawberry [15]. These compounds can be extracted from natural sources. However, they might be contaminated by other complex compounds during extraction. In addition, it is difficult to meet the increasing demand by extraction from natural sources because desired substances are present in very low concentrations [16]. Although chemical production methods allow inexpensive and rapid mass synthesis, they are not environmentally friendly due to the use of very high temperatures and strong bases or acids [17, 18]. For food ingredients, the demand for environmentally friendly products is greatly increasing and the method of using a biocatalyst is attracting the attention of many researchers.

Recently, experiments on ester synthesis using cutinases derived from several microorganisms such as *T. fusca*, *B. cepacia*, *Thielavia terrestris*, *Fusarium solani pisi*, *Fusarium oxysporum*, and *Malbranchea cinnamomea* have been conducted [7, 8, 19-22]. In a previous study, we have obtained cutinase (*R*cut) from *Rhodococcus* strains (RosL12) and investigated their properties for hydrolysis, polymer degradation, and transesterification reactions [23]. In this study, we used immobilized *R*cut (imm*R*cut) as a biocatalyst to produce short/medium chain esters that could be used as food additives. The substrate specificity of *R*cut was analyzed through molecular docking.

## Material and Methods

### Chemicals and Materials

Ethyl butyrate, butyl butyrate, hexyl butyrate, and butyric acid were purchased from Tokyo Chemical Industry. n-Octyl butyrate was purchased from Thermo Fisher Scientific. Molecular sieve 4 Å, ethanol, 1-hexanol, 1-octanol, 1-decanol, p-nitrophenyl caprylate (pNPC), and glutaraldehyde solution were purchased from Sigma-Aldrich. 1-Butanol, n-hexane, n-heptane, cyclohexane, and isooctane were purchased from Samchun. Methacrylate-divinylbenzene (MA-DVB) resin was purchased from GenoFocus.

### Enzyme Expression and Immobilization

The pET22 vector containing the cutinase gene (*R*cut) [23] of *Rhodococcus* sp. was transformed into *Escherichia coli* Rosetta-gami 2 (DE3) pLysS. The transformant was cultured in Luria-Bertani (LB) broth (200 ml) and the recombinant cutinase was overexpressed using 1 mM isopropyl β-D-1-thiogalactopyranoside (IPTG). After *E. coli* cells were harvested and disrupted by sonication, 10 ml of cell free extract (CFE) (36 mg) was obtained. Methacrylate-divinylbenzene (MA-DVB) resin (1 g) was washed with methanol (10 ml) at 150 rpm for 1 h. Methanol was removed and the resin was washed three times with Tris-HCl buffer (pH 8.0). After washing buffer was removed, resin, 10 ml of CFE, and glutaraldehyde (final concentration 25 mM) were incubated at 4°C for 20 h with inverting. The resin on which the CFE was immobilized was washed three times with Tris-HCl buffer. The remaining solvent was removed with a speed vac evaporator.

### Hydrolysis Activity Assay of Cell Free Extract and Immobilized *R*cut

Hydrolysis activity assay was performed using p-nitrophenyl caprylate (pNPC) as a substrate. The enzyme reaction was performed in a 1 ml reaction mixture containing 950 μl of 50 mM Tris-HCl buffer (pH 8.0), 40 μl of ethanol, and 10 μl of 10 mM pNPC dissolved in acetonitrile. After adding 10 μl of CFE, the reaction mixture was incubated at 30°C for 3 min. The concentration of p-nitrophenol was determined by measuring the absorbance at 405 nm. One unit of enzyme activity was defined as 1 μmol of p-nitrophenol released per min under assay conditions. Enzyme activity was measured in triplicate and the average value was calculated. The hydrolysis reaction was performed under the same conditions as the free enzyme using 5 mg of the immobilized enzyme. One unit of immobilized enzyme activity was defined as 1 μmol of p-nitrophenol released per min under assay conditions.

### High-Performance Liquid Chromatography (HPLC) Analysis

Cogent Bidentate C18 column (4.6 mm × 250 mm, 5 μm particle size; microSolv Technology Corp., USA) was used and 10 μl of the reaction sample was injected. Elution was performed at 30°C using a 9:1 ratio of acetonitrile and water at a flow rate of 1 ml/min. Eluted material was analyzed with a refractive index detector (Agilent 1100 HPLC G1362A) at 35°C. Fig. S1 shows results of HPLC analysis for each alkyl butyrate synthesized in reactions.

### Effect of Temperature on Butyl Butyrate (BB) Synthesis with Imm*R*cut

Esterification reaction was carried out by adding 100 mM butyric acid, 100 mM 1-butanol, and 100 mg imm*R*cut to 5 ml of isooctane solvent in a 20 ml screw vial. To investigate changes in enzyme activity according to temperature, the BB synthesis reaction was performed at each temperature of 30, 40, and 50°C, respectively. The experiment was performed in duplicate. After the reaction, the synthesized BB was analyzed by HPLC. The peak area of BB was calculated by comparing it with the authentic BB.

### BB synthesis according to Concentrations of Butyric Acid and 1-Butanol

To investigate the synthesis of BB according to the concentration of butyric acid, 50 mM to 250 mM (at 50 mM intervals) butyric acid and 100 mM 1-butanol were added to 5 ml of isooctane solvent in a 20 ml screw vial. BB synthesis reaction was performed in duplicate at 30°C for 24 h at 140 rpm with 20 mg of imm*R*cut.

To investigate the synthesis of BB according to the concentration of 1-butanol, 100 mM butyric acid and 50 mM to 250 mM 1-butanol (at 50 mM intervals) were added under the same conditions by changing the substrate concentration. The reaction was performed in duplicate at 30°C for 24 h at 140 rpm. The synthesized BB was analyzed by HPLC.

### Effect of Organic Solvents on Imm*R*cut for BB Synthesis

To investigate the synthesis of BB according to organic solvent, 100 mM butyric acid and 100 mM 1-butanol were added to 5 ml of each solvent (isooctane, heptane, hexane, and cyclohexane) in a 20 ml screw vial. BB synthesis reaction was performed with 20 mg of imm*R*cut. The reaction was performed in duplicate at 30°C for 24 h at 140 rpm. The BB synthesized by reaction in each solvent was then analyzed by HPLC.

### Investigating the Stability of Imm*R*cut according to the Number of Reuses and Continuous BB Synthesis Using Imm*R*cut

To investigate the stability according to the number of reuses of the imm*R*cut, 100 mM butyric acid and 100 mM 1-butanol were added to 5 ml of isooctane solvent in a 20 ml screw vial. BB synthesis reaction was performed with 100 mg of imm*R*cut and 400 mg of molecular sieve. The reaction was performed in duplicate at 30°C for 24 h at 140 rpm. After the reaction, the imm*R*cut was washed three times using 5 ml of isooctane solvent. The synthesis reaction was repeated seven times under the same conditions. The BB synthesized in the initial reaction and the BB synthesized according to the number of reuses were compared.

To investigate the continuous synthesis of BB with the imm*R*cut, 100 mM butyric acid and 100 mM 1-butanol were added to 5 ml of isooctane solvent in a 20 ml screw vial. BB synthesis reaction was performed with 100 mg of imm*R*cut and 400 mg of molecular sieve. The reaction was performed in duplicate at 30°C for 24 h at 140 rpm. After the reaction, 100 mM butyric acid and 1-butanol were added every day and the synthesis reaction was continued for four days. In another reaction, the synthesis reaction was performed under the same conditions without the addition of a substrate at an initial substrate concentration of 500 mM butyric acid and 1-butanol. The BB synthesized in reaction was analyzed by HPLC.

### Investigating Alcohol Preference of Imm*R*cut for the synthesis of alkyl butyrate

To investigate the preferred alcohol in the synthesis of alkyl butyrate, butyric acid and various alcohol (ethanol, 1-butanol, 1-hexanol, 1-octanol, and 1-decanol) were used. Briefly, 100 mM butyric acid and 100 mM alcohol were reacted with 100 mg of imm*R*cut, respectively. The reaction was performed in duplicate at 30°C for 8 h at 140 rpm. The synthesized alkyl butyrate was analyzed by HPLC. Synthesized amounts of ethyl butyrate, BB, hexyl butyrate, and octyl butyrate were compared with purchased standard compounds. For decyl butyrate, which could not be purchased, the amount of decyl butyrate synthesized was measured as the amount of 1-decanol decreased after the synthesis reaction. Fig. S2 shows the equation for calculating the concentration of each synthesized alkyl butyrate.

### In silico Molecular Docking Analysis Using Autodock

Autodock 4.2.6 and MGLTools 1.5.7 version (https://ccsb.scripps.edu/) were used for molecular docking analysis. The 3D structure of *R*cut was made with *F. solani* cutinase (PDB: 1CEX) as a template using amino acid sequences of *R*cut. 3D modeling was performed in SWISS-MODEL (https://swissmodel.expasy.org/) [24]. 3D models of ethanol, 1-butanol, 1-hexanol, 1-octanol, and 1-decanol were downloaded from https://pubchem.ncbi.nlm.nih.gov/. Protein-ligand molecule docking was performed according to the tutorial of AutodockTools. Grid box coordinates were set to be: X, 7.5; Y, 55.8; and Z, 17.5. Spacing (Å) was set to 0.250. Genetic algorithm parameters were set as basic settings (number of GA run: 10; population size: 150). The docking output was set to lamarckian GA (4.2).

## Results and Discussion

### Production and Immobilization of *R*cut

*E. coli* Rosetta-gami 2 (DE3) pLysS containing *R*cut gene was cultured and recombinant *R*cut was produced by IPTG induction. CFE was then prepared by disrupting *E. coli* cells using sonication. The hydrolytic activity of CFE was determined using a pNPC substrate. It was measured to be 25.7 U/mg protein. To synthesize ester compounds in a non-aqueous system, *R*cut was immobilized on MA-DVB resin using adsorption and cross-linking with glutaraldehyde. A total of 36 mg protein (10 ml) of CFE was used for protein immobilization. The amount of unbound protein was 6 mg. Thus, the immobilization efficiency was calculated to be 83%. The hydrolytic activity of immobilized *R*cut was measured to be 1.26 U/g and the activity retention was calculated to be 0.14% (Table 1). Activity retention was measured to be very low due to the difference between the two assay systems. The assay using CFE was a homogenous system. However, the assay using an immobilized enzyme was a heterogeneous system. That is, in a heterogeneous system, the reaction rate was greatly reduced because the substrate concentration close to the enzyme was very low. However, since the immobilized enzyme must be used to perform the ester synthesis reaction in a non-aqueous system, we tested whether our immobilized enzyme could perform the desired synthesis reaction.

### Effect of Temperature on Synthesis of BB Using Imm*R*cut

**Scheme 1:**
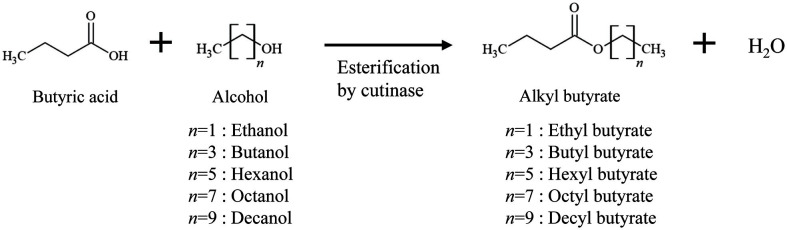
Schematic depiction of alkyl butyrate synthesis by esterification reaction using immobilized cutinase (imm*R*cut).

We determined whether *R*cut immobilized on MA-DVB beads could synthesize BB using butyric acid and butanol as substrates (Scheme 1). As a result of performing the reaction in a non-aqueous system (isooctane solvent) followed by analysis by HPLC, it was confirmed that BB was synthesized (Fig. S1).

**Fig. 1:**
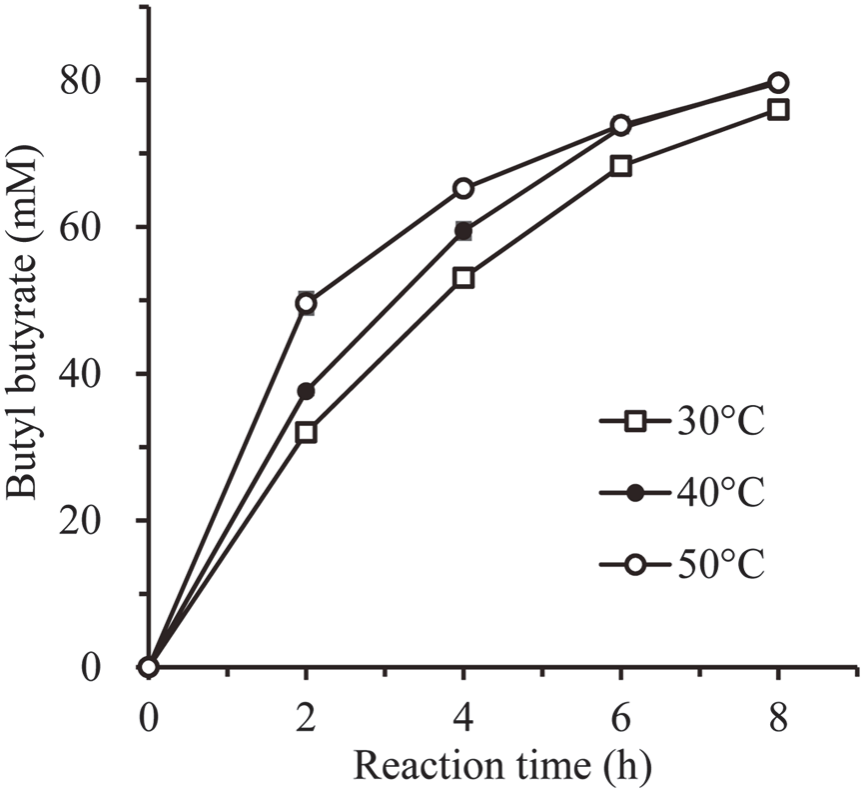
Effect of temperature on butyl butyrate synthesis with imm*R*cut. Synthesis reaction was performed at each temperature using 100 mM butyric acid and 100 mM 1-butanol as substrates.

To characterize the enzymatic reaction, the optimal reaction temperature was first investigated. The amount of BB produced was measured while the reaction was carried out at 30, 40, and 50°C. Fig. 1 shows the synthesis efficiency of BB at each temperature with a time course. Although there was a difference in BB synthesis efficiency at the initial stage (2-6 h), when the reaction was carried out for 8 h, the synthesis efficiency at each temperature did not show a significant difference. Approximately 80 mM of BB was produced after 8 h of reaction. Thus, we set the reaction temperature for subsequent reactions to 30°C because the substrate and reaction products evaporated at higher temperatures.

On the other hand, when the enzyme was immobilized, it was known to have better stability in organic solvents and at high temperatures [25]. We confirmed that the temperature stability of *R*cut was increased by immobilizing it on the beads. In a previous study, a free *R*cut showed 25% residual activity after treatment at 40°C for 2 h [23]. It lost almost all of its activity after treatment at 50°C for only 10 min. However, as shown in Fig. 1, imm*R*cut showed significantly increased thermal stability even at 50°C. Therefore, imm*R*cut is expected to be effectively used in various reactions to produce heat-stable substrates and reaction products.

### Effect of Butyric Acid and 1-Butanol Concentrations on BB Synthesis with Imm*R*cut

Since the production yield of the esterification reaction varied depending on the concentration of the substrate used in the reaction, the production of BB was measured while changing concentrations of butyric acid and butanol in the reaction solution.

**Fig. 2:**
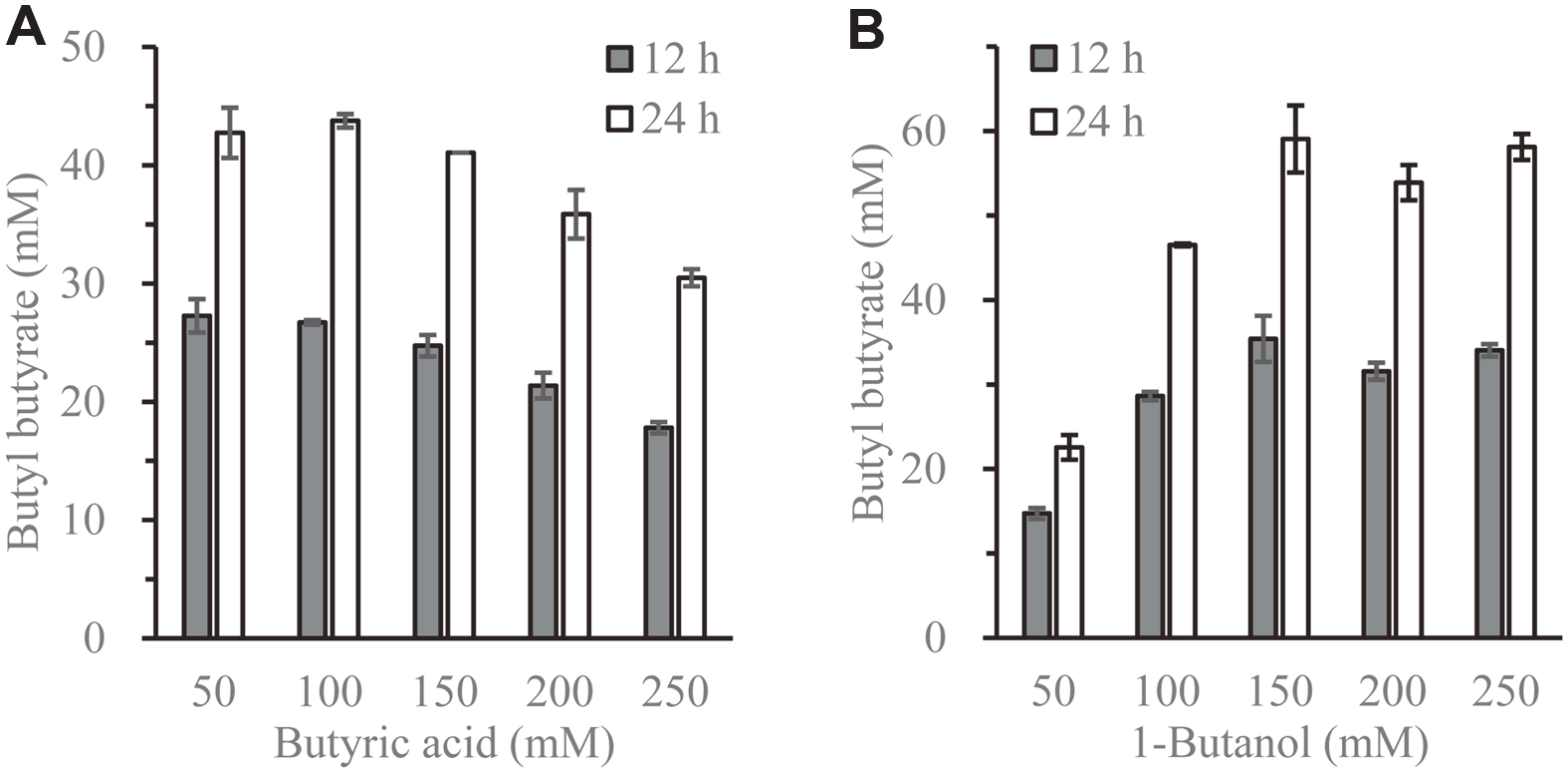
Effects of butyric acid and 1-butanol concentrations on butyl butyrate synthesis using imm*R*cut. (**A**) The amount of BB synthesized was measured when the 1-butanol concentration was fixed at 100 mM and the butyric acid concentration was increased from 50 to 250 mM. (**B**) The synthesis amount of BB was measured when the butytic acid concentration was fixed at 100 mM and the 1-butanol concentration was increased from 50 to 250 mM.

Fig. 2A shows BB production according to the concentration of butyric acid with the concentration of butanol fixed at 100 mM. After 12 h reaction, 27 mM of BB was produced with 50 mM or 100 mM butyric acid. BB concentration was decreased when the concentration of butyric acid was over 150 mM. After 24 h reaction, 43 mM of BB was produced with 50 or 100 mM butyric acid. BB concentration was also decreased when butyric acid concentration was over 150 mM. Butyric acid acted as both a substrate and a solvent. At the beginning of the reaction, there was little water in the reaction solution. However, when water was generated as esterification reaction proceeded, butyric acid lowered the pH of the reaction solution, thus decreasing the enzyme activity.

Fig. 2B shows BB production according to the concentration of 1-butanol with the concentration of butyric acid fixed at 100 mM. After 12 h reaction, BB concentration increased, reaching 45 mM when butanol concentration was 150 mM. No noticeable increase in BB concentration was observed when butanol concentration was increased up to 250 mM. After 24 h reaction, BB concentration increased up to 60 mM with 150 mM butanol. It showed no noticeable increase when butanol concentration was increased up to 250 mM. It was found that butanol did not adversely affect the enzyme activity even at high concentrations (up to 250 mM, 1.85%), unlike butyric acid. These results indicate that appropriate concentrations of butyric acid and 1-butanol are important for efficient BB synthesis with imm*R*cut.

Several papers have reported the effect of substrate concentration on esterification reactions. *T. terrestris* CAU709 cutinase showed the highest esterification efficiency with 120 mM butyric acid and 80 mM 1-butanol [22]. The synthesis reaction of BB using *Thermomyces lanuginosus* lipase showed the best efficiency with 100 mM butyric acid and 300 mM 1-butanol [26]. Alkaline lipase derived from *Acinetobacter* Sp. EH28 showed the highest esterification efficiency with 130 mM caprylic acid and 100 mM ethanol [27]. Thus, the concentration of the substrate to efficiently synthesize the ester compound seems to be different for each enzyme. In our subsequent experiment, to investigate other properties of imm*R*cut, a synthesis reaction was performed at a substrate concentration of 100 mM.

### Effects of Organic Solvents on the Synthesis of BB with Imm*R*cut

**Fig. 3:**
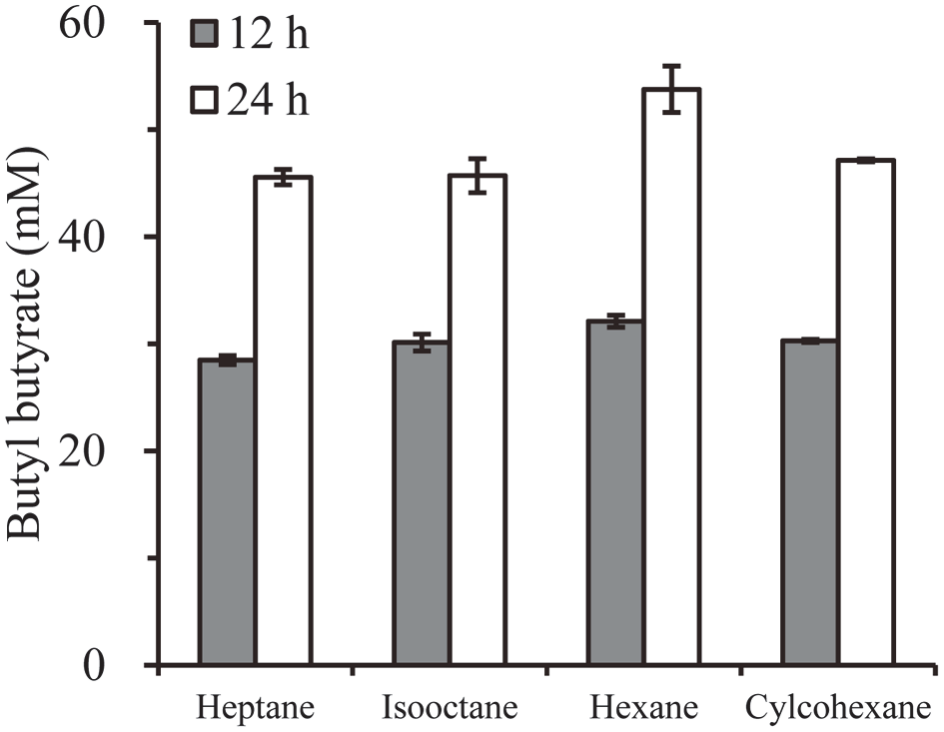
Effects of organic solvents on the synthesis of butyl butyrate. The BB synthesis reaction in each solvent was performed using 100 mM butyric acid and 100 mM 1-butanol as substrates.

To investigate the BB synthesis efficiency of the imm*R*cut according to organic solvents, BB was synthesized in four different non-polar solvents (Fig. 3). Hexane (logP 4.6), cyclohexane (logP 4.3), isooctane (logP 3.9), and heptane (logP 3.4) can dissolve both butyric acid and butanol. Thus, they were used as reaction solvents. After 12 h reaction, BB concentration reached about 30 mM in all solvent systems. After 24 h reaction, BB concentration increased further up to 45-50 mM. Slightly better enzyme activity was observed in hexane than in the other three solvents. The vapor pressure of hexane was higher than those of the other solvents: hexane (120 mmHg, 20°C), cyclohexane (95 mmHg, 20°C), isooctane (40 mmHg, 21°C), and heptane (40 mmHg, 22°C) [28]. However, it was difficult to find an evident relationship between the vapor pressure of the solvent and BB production. In previous studies, when three types of cutinase were immobilized, there were no significant differences in their activities in organic solvents exceeding the logP value of 1.5 [29]. On the other hand, it is disadvantageous to use a solvent having a high vapor pressure because the reaction volume decreases when the reaction is performed for a long time. Therefore, our subsequent experiments were performed in isooctane solvent having a low vapor pressure.

### Stability of Imm*R*cut according to the Number of Reuses and Continuous Synthesis Reaction

**Fig. 4:**
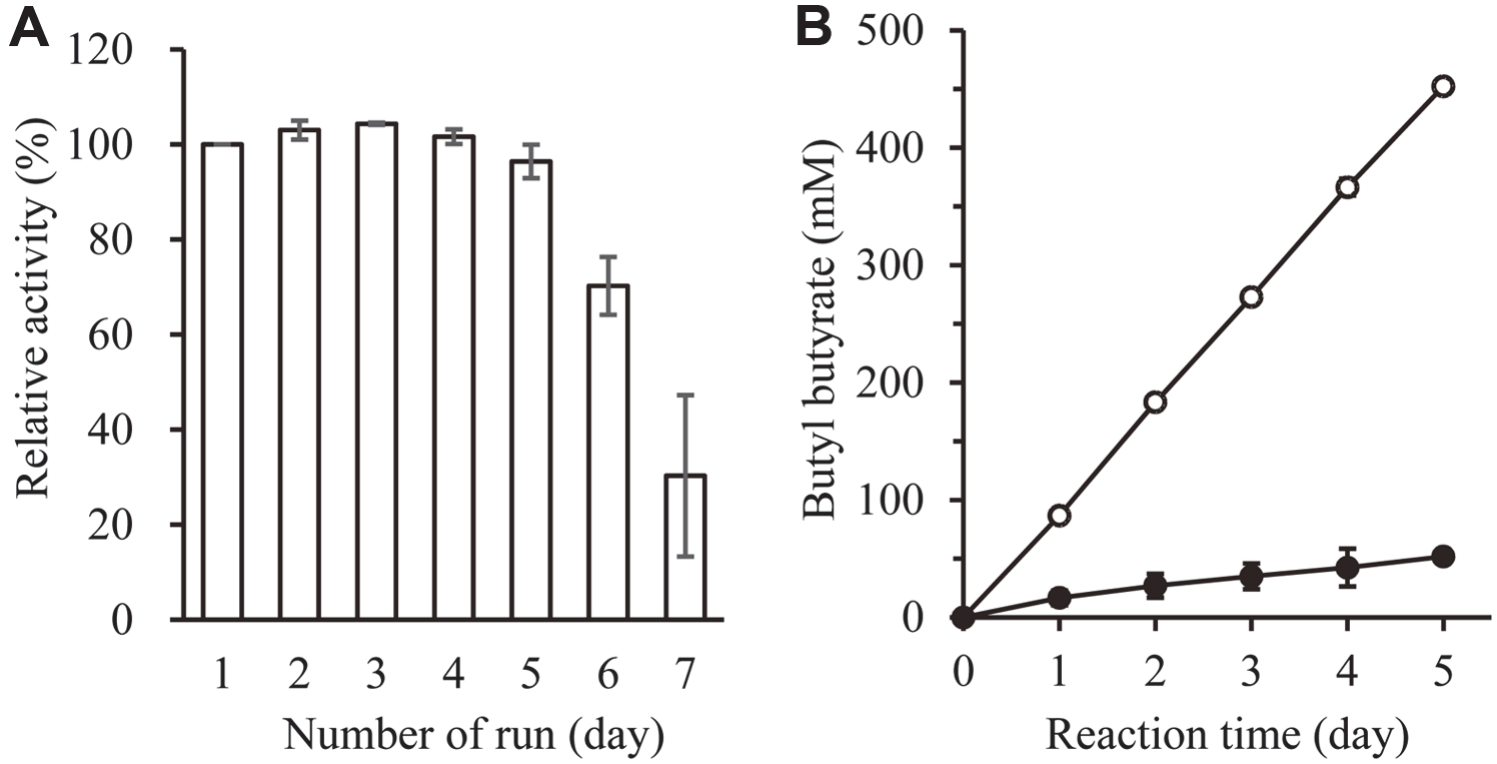
Investigation of the stability of imm*R*cut in isooctane solvent and continuous synthesis of butyl butyrate using imm*R*cut. (**A**) The reaction was performed in isoctane solvent with 100 mM butyric acid and 100 mM 1- butanol as substrates. After washing the used immobilized enzyme, the reaction was repeated 7 times under the same conditions. (**B**) BB was synthesized continuously for 5 days using imm*R*cut. Butyric acid and 1-butanol at 100 mM were added to the reaction solution every 24 h (open circle). Butyric acid and 1-butanol at 500 mM were added at the beginning of the reaction (closed circle).

To investigate how long imm*R*cut could maintain its enzyme activity, the reaction was repeated under the same reaction condition for 7 days. As shown in Fig. 4A, the initial synthesis efficiency was maintained in isooctane solvent until the 5^th^ day and the synthesis efficiency decreased from the 6^th^ day. In previous studies, *F. solani* cutinase (FsC) immobilized on magnetic genipin-crosslinked chitosan beads showed reduced activity by 70%after reuse for the 5^th^ time [30]. Cross-linked enzyme aggregates (CLEAs) prepared using *F. oxysporum* cutinase (FoC) showed a decrease in the activity of up to 60% at the 4^th^ reuse [21]. Imm*R*cut was proven to have the ability to maintain enzyme activity up to the 5^th^ day. This suggests that imm*R*cut has value for industrial applications.

To mass produce BB, 100 mM butyric acid and butanol were added daily for 5 days. As shown in Fig. 4B, BB production continued to increase with 452 mM BB produced after 5 days. This provided evidence that the enzyme activity did not decrease for 5 days and that the synthesis reaction was performed stably. On the other hand, when 500 mM butyric acid and butanol were added at once at the beginning and the reaction was performed, the amount of BB was only 52 mM after 5 days. This was because the enzyme activity decreased when the butyric acid concentration was increased to 150 mM or more as shown in Fig. 2A. Therefore, to synthesize a high concentration of BB, it is necessary to add the substrate several times at a concentration of 100 mM.

### Alkyl Butyrate Synthesis of Imm*R*cut according to the Chain Length of Alcohol

**Fig. 5:**
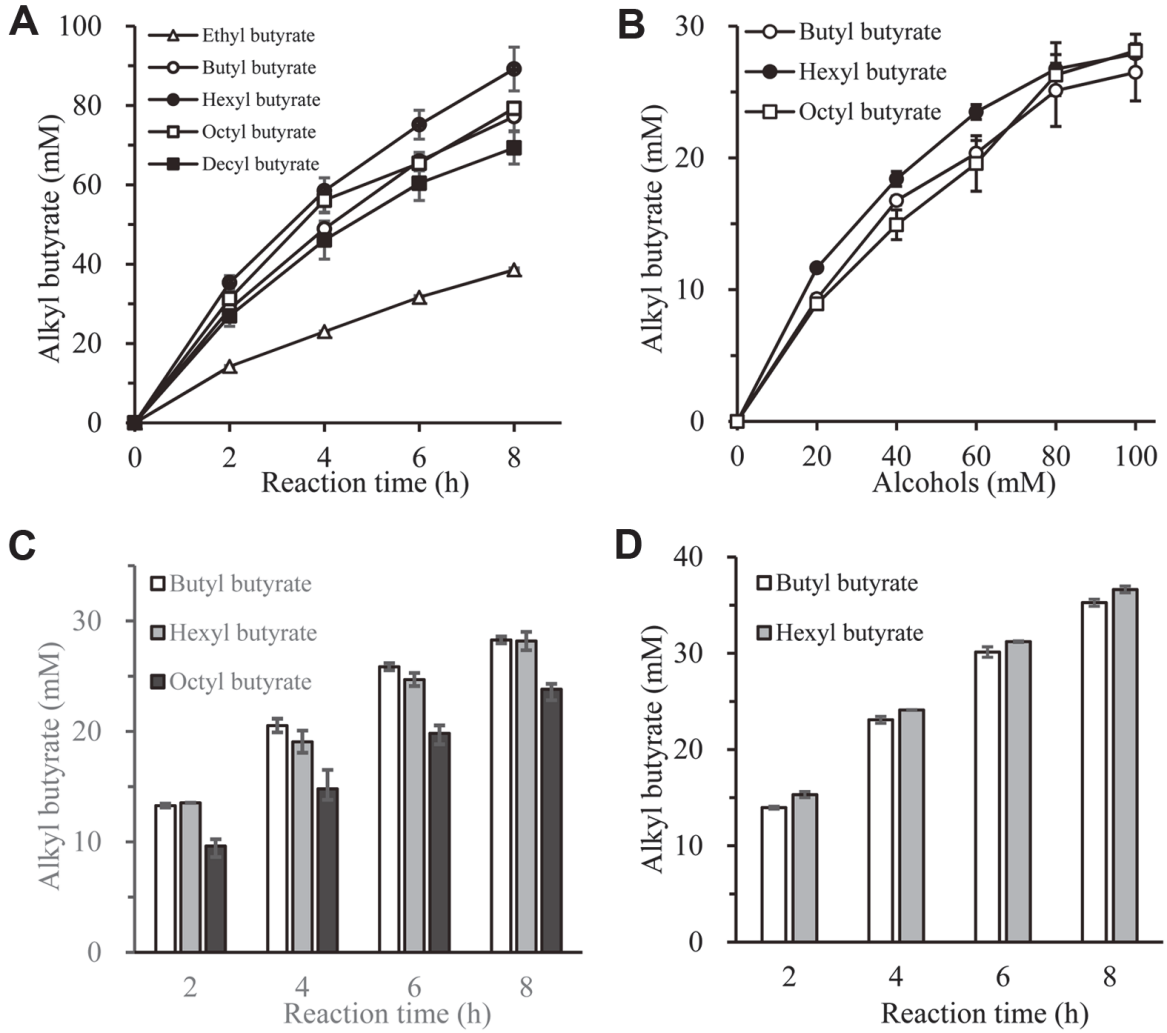
Alkyl butyrate synthesis of imm*R*cut according to the chain length of alcohol. (**A**) Each reaction was carried out with 100 mM of butyric acid and 100 mM of each alcohol (ethanol, 1-butanol, 1-hexanol, 1-octanol, 1-decanol) as substrates. (**B**) The synthesis amount of alkyl butyrate was measured while the butytic acid concentration was fixed at 100 mM and the alcohol (1-butanol, 1-hexanol, 1-octanol) concentration was increased from 0 to 100 mM. (**C**) 100 mM butyric acid, 50 mM 1-butanol, 50 mM 1-hexanol, and 50 mM 1-octanol were all put into one vial and the synthesis reaction was performed. (**D**) 100 mM butyric acid, 50 mM 1-butanol, and 50 mM 1-hexanol were all put into one vial and the synthesis reaction was performed.

Since various alkyl esters as well as BB have been used in the food industry, ester synthesis was performed using various alcohols and butyric acid as substrates (Scheme 1). Reaction for synthesizing alkyl butyrate was performed using 100 mM butyric acid and 100 mM of various alcohol (C2-C10). As shown in Fig. 5A, the highest enzyme activity was observed with C6 chain substrate, with 85 mM hexyl butyrate produced within 8 h. Octyl butyrate, BB, and decyl butyrate were also made in large quantities in that order. Ethyl butyrate was the least produced.

In a previous study on esterification reaction using *F. solani* *pisi* cutinase, C4-C6 substrates showed the highest synthesis efficiency [19]. In the case of *F. oxysporum* cutinase, C4 showed the highest efficiency when an alkyl butyrate was synthesized by a transesterification reaction [21]. Esterification reaction using *T. terrestris* cutinase showed the highest efficiency when synthesizing hexyl hexanoate [31]. In the alkyl butyrate synthesis reaction using *B. cepacia* NRRL B 2320 cutinase, C4-C6 alcohol showed high efficiency [8]. As such, the high substrate specificity of C4-C6 seems to be a typical characteristic of cutinase.

Synthesis efficiency was measured while increasing concentrations of C4, C6, and C8 substrates (Fig. 5B). At a high concentration (80 or 100 mM), no significant difference was observed for different substrates. However, at a low concentration (20, 40, or 60 mM), the C6 substrate had the best synthesis efficiency.

To accurately investigate substrate preference, three alcohols were simultaneously put into one vial and the production of each product was measured. The reaction of synthesizing the product with C4, C6, and C8 substrates was performed (Fig. 5C). As a result of the reaction, it was observed that C4 and C6 had distinctly higher synthesis efficiencies than C8 substrate. In order to observe the substrate preference more clearly, the synthesis reaction was performed with two substrates (C4 and C6) in one vial (Fig. 5D). When the two substrates were present, C6 showed better synthesis efficiency than C4. These results revealed that imm*R*cut preferred C4-C8 substrates, showing a good synthesis efficiency in the order of C6 > C4 > C8 > C10 > C2.

### In silico Molecular Docking Analysis for Substrate Specificity Using Autodock

**Fig. 6:**
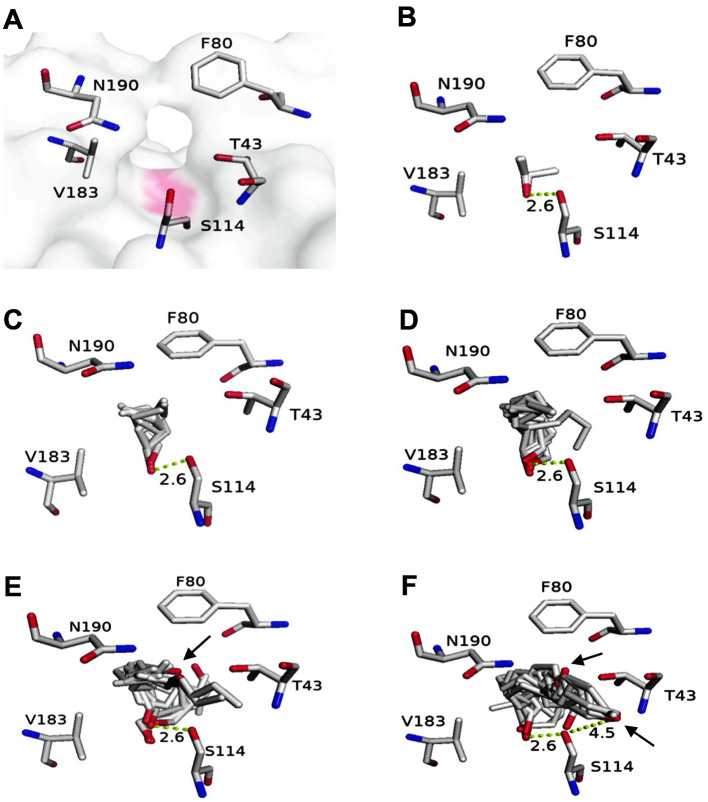
3D structure of the active site of *R*cut and the result of molecular docking of each alcohol and the active site of *R*cut. (**A**) Amino acids near the active site of *R*cut are indicated. S114 is responsible for the enzymatic activity of *R*cut. Results of molecular docking of 10 volunteer ethanol (**B**), 1-butanol (**C**), 1-hexanol (**D**), 1-ocatanol (**E**), and 1-decanol (**F**) to the active site of the enzyme are presented. In the molecular structure, arrows indicate hydroxyl groups of each alcohol.

Molecular docking analysis was performed to explain the substrate specificity of *R*cut in alkyl butyrate synthesis. Fig. 6A shows amino acids near the active site of *R*cut. S114 is the active site serine. N190 and F80 appear to be gatekeepers through which the substrate can access the enzyme pocket. When the accessibility of each alcohol to the active site of the enzyme was investigated through molecular docking, the complex of *R*cut and substrate with the longest carbon chain (1-decanol) had the lowest free energy (-4.58 kcal/mol) (Table 2). It has been reported that hydrophobic aromatic residues near the active site of the enzyme might be related to the substrate specificity of the long carbon chain [32]. The reason why the most hydrophobic alcohol has the highest binding energy might be due to the protruding phenyl group of F80 among amino acids that can act as gatekeepers.

In the esterification reaction, according to the ping-pong bibi mechanism, an acid and an enzyme can combine to form an acyl intermediate. The intermediate is again converted into an ester and an enzyme by a nucleophilic attack of the hydroxyl group of the alcohol [33]. That is, the accessibility of alcohol to the enzyme can affect the esterification reaction. However, according to our results, the accessibility of these alcohols and the substrate preference of the enzyme did not exactly match (Fig. 5). 1-Decanol was expected to be the most efficient one. However, the highest efficiency was shown in 1-hexanol with 6 carbon chains.

Figs. 6B-6F show the results of molecular docking using each alcohol and enzyme. Hydroxyl groups are shown in red. In ethanol (Fig. 6B), 1-butanol (Fig. 6C), and 1-hexanol (Fig. 6D), the hydroxyl oxygen of alcohol was neatly aligned near the ^γ^O-S114. However, in 1-octanol (Fig. 6E) and 1-decanol (Fig. 6F), the hydroxyl oxygen did not align with the ^γ^O-S114. In addition, hydroxyl groups of 1-decanol generated more volunteers than those of 1-octanol, which aligned in a different direction from the ^γ^O-S114. In addition, when the average distance between the hydroxyl oxygen of each alcohol and the ^γ^O-S114 was measured, it was observed that the distance increased as the carbon length of the alcohol increased. In the case of 1-decanol, the distance was much longer than 3 Å (Table 2). This phenomenon appeared to be caused by the torsion number of alcohol. That is, as the carbon length increased, the torsion number increased. The increase in torsion number seems to prevent hydroxyl oxygen of the alcohol from performing the esterification reaction.

When the substrate specificity of *Aspergillus niger* lipase (EXANL1) was analyzed through molecular docking, the binding free energy was important. However, the arrangement of active site serine and substrate was much more important for substrate specificity [34]. Based on these results, we can conclude that among alcohols that are properly aligned with the active site Ser, 1-hexanol seems to show the best efficiency for the synthesis of alkyl butyrate.

Unlike *R*cut, when synthesizing alkyl butyrate using *F. oxysporum* cutinase (FoC), 1-butanol showed the highest synthesis efficiency, while alcohol with a longer carbon chain showed a gradual decrease in synthesis efficiency [21]. FoC was also analyzed through molecular docking (Fig. S3). The hydroxyl oxygen of 1-butanol was neatly aligned in the ^γ^O of Ser at the active site. However, FoC showed that some volunteers of 1-hexanol were not aligned with the ^γ^O of Ser at the active site, which was different from the case of *R*cut. This discrepancy was more evident in 1-heptanol with two more carbons. These results suggest that whether the hydroxyl oxygen of alcohol correctly aligns with the active site Ser determines the substrate specificity in the synthesis of alkyl butyrate.

In this study, alkyl butyrate useful as a food additive was synthesized by performing an esterification reaction using imm*R*cut. As a result of testing the synthesis efficiency according to substrate concentration, butyric acid showed a high synthesis efficiency at a concentration of 100 mM while butanol showed a high synthesis efficiency at a concentration of 150 mM or higher. Imm*R*cut was able to perform synthetic reactions stably in non-polar organic solvents. When an alkyl butyrate was synthesized using alcohol having a C2-C10 carbon chain, imm*R*cut preferred C4-C8 substrate and showed a good synthesis efficiency in the order of C6 > C4 > C8 > C10 > C2. As a result of molecular docking analysis, it was confirmed that the long-chain substrate had a high binding energy, but was difficult to properly align to the active site of the enzyme due to torsion. Therefore, it is expected that C6, which is properly aligned with a sufficiently high binding energy, has the highest synthesis efficiency in the synthesis of alkyl butyrate. High activity was maintained up to 5 times when imm*R*cut was recycled and BB was continuously synthesized for 5 days when the substrate was added to the vial daily. This study structurally elucidated the substrate specificity of the enzyme. The results of this study suggest that *R*cut can be used industrially.

## Supplemental Materials

Supplementary data for this paper are available on-line only at http://jmb.or.kr.

## Figures and Tables

**Table 1:** Enzyme activity of cell free extract and imm*R*cut.

	Enzyme activity	Total weight (mg)	Total activity (U)
Cell Free Extract	25.7 U/mg	36	925
Imm*R*cut	1.26 U/g	1000	1.26

**Table 2:** Chemical property of alcohol, binding energy, and average distance.

	Number of carbons	Number of rotable bonds	Binding energy (kcal/mol)	Average distance (Å)*
Ethanol	2	1	-2.78	-
1-butanol	4	3	-3.39	2.6
1-hexanol	6	5	-3.83	2.6
1-octanol	8	7	-4.34	2.9
1-decanol	10	9	-4.58	3.6

*Average distance between the hydroxyl group of 50 each alcohol molecules and the oxygen of the active site serine of the enzyme.
