# Supplementary material for: Synthesis of Short-Chain Alkyl Butyrate through Esterification Reaction Using Immobilized Rhodococcus Cutinase and Analysis of Substrate Specificity through Molecular Docking
Source: J Microbiol Biotechnol. 2022 Dec 2;33(2):268–76. doi: 10.4014/jmb.2211.11022 (PMC9998203; doi:10.4014/jmb.2211.11022)

J. Microbiol. Biotechnol.
https://doi.org/10.4014/jmb.2211.11022

Fig. S1. HPLC analysis of ethyl butyrate (A)

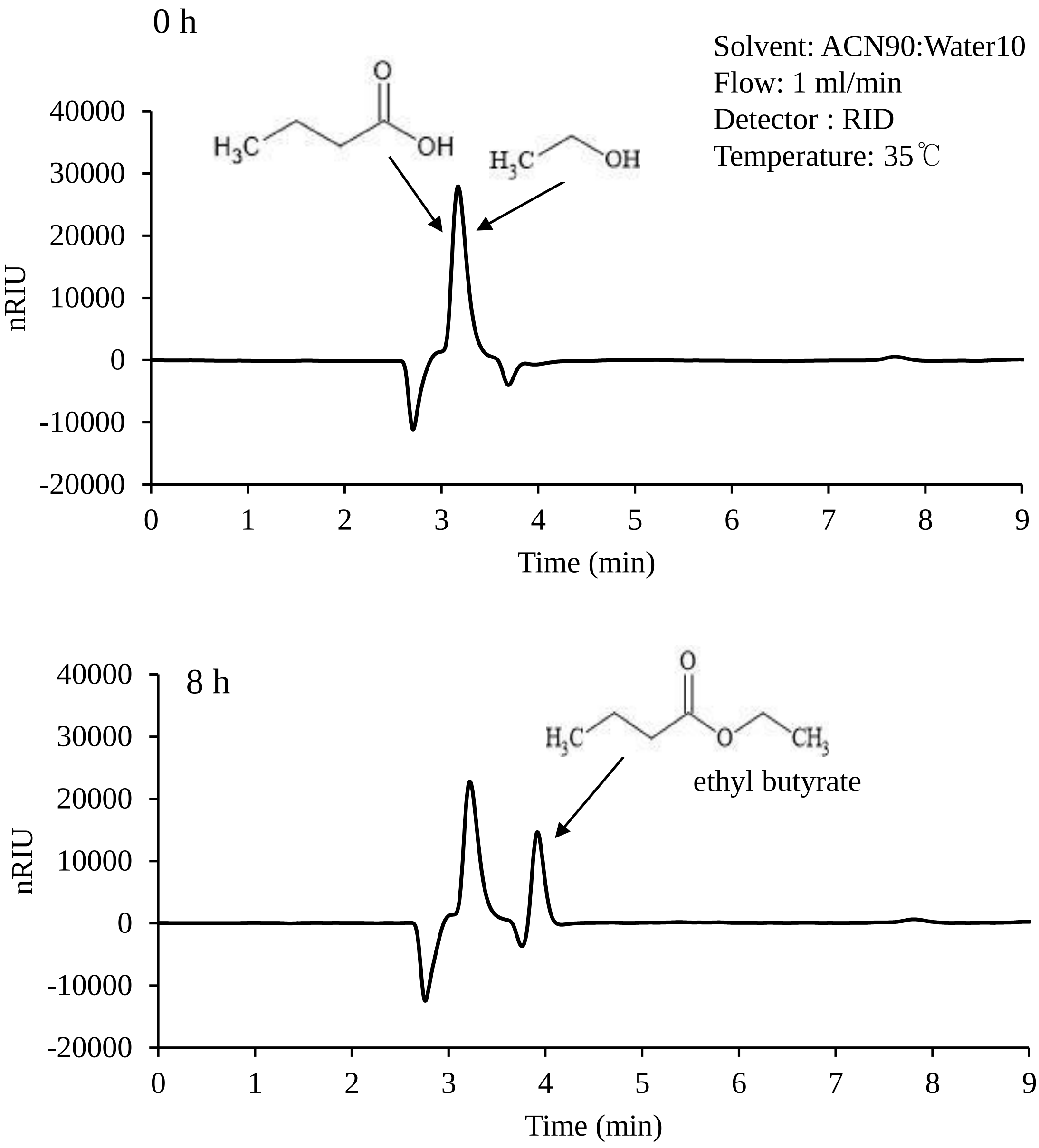

Fig. S1. HPLC analysis of butyl butyrate (B)

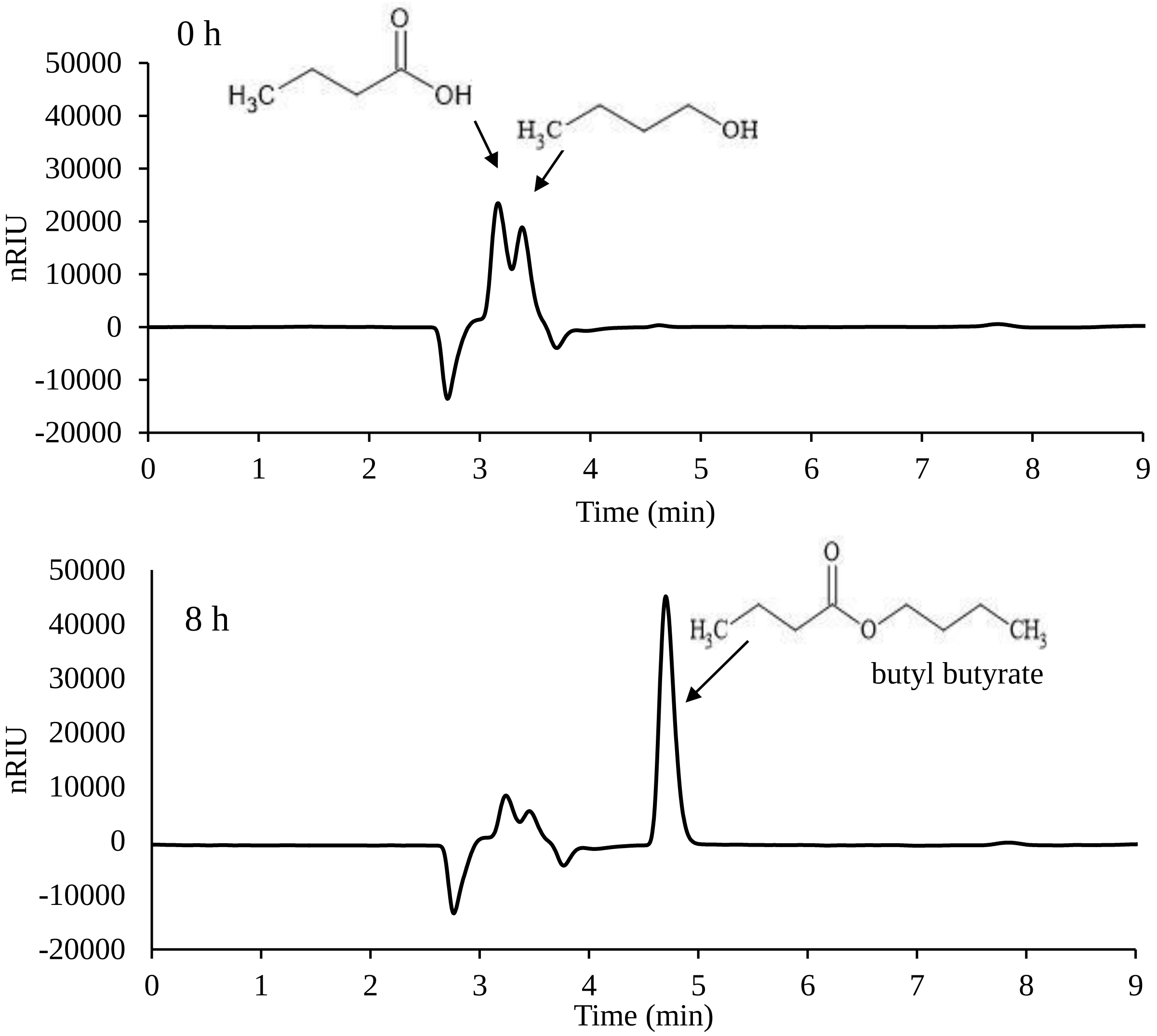

Fig. S1. HPLC analysis of hexyl butyrate (C)

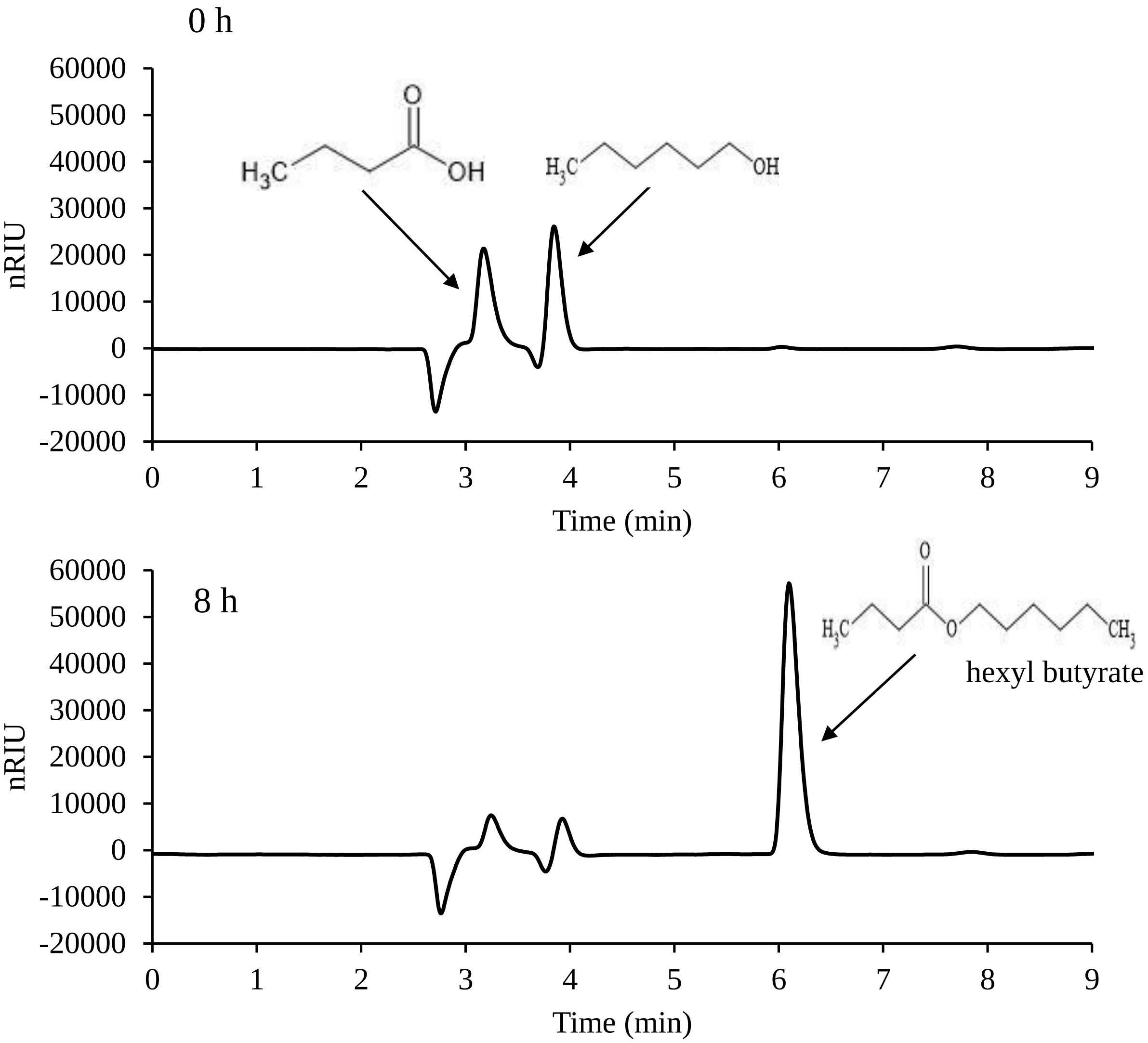

Fig. S1. HPLC analysis of octyl butyrate (D)

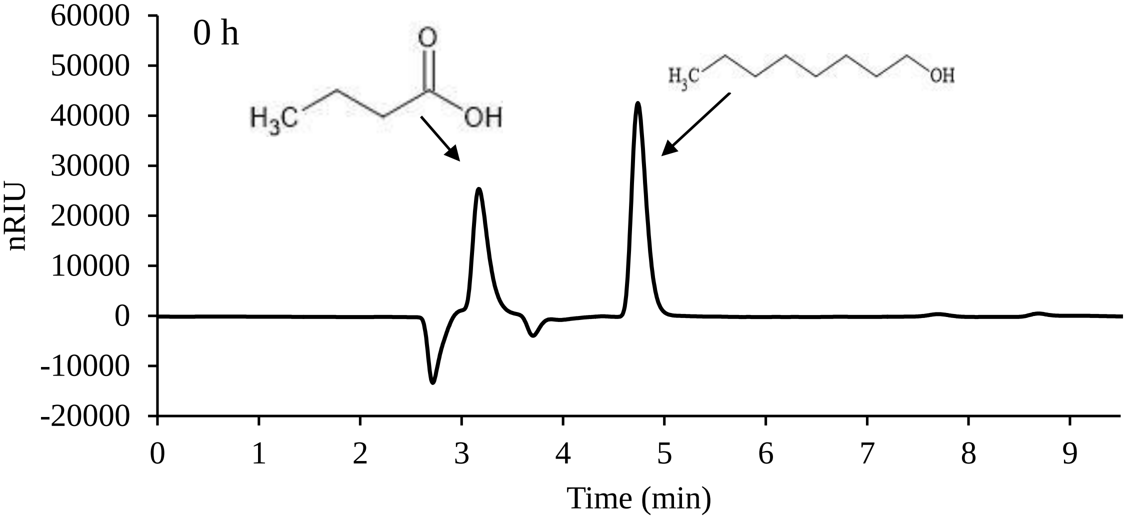

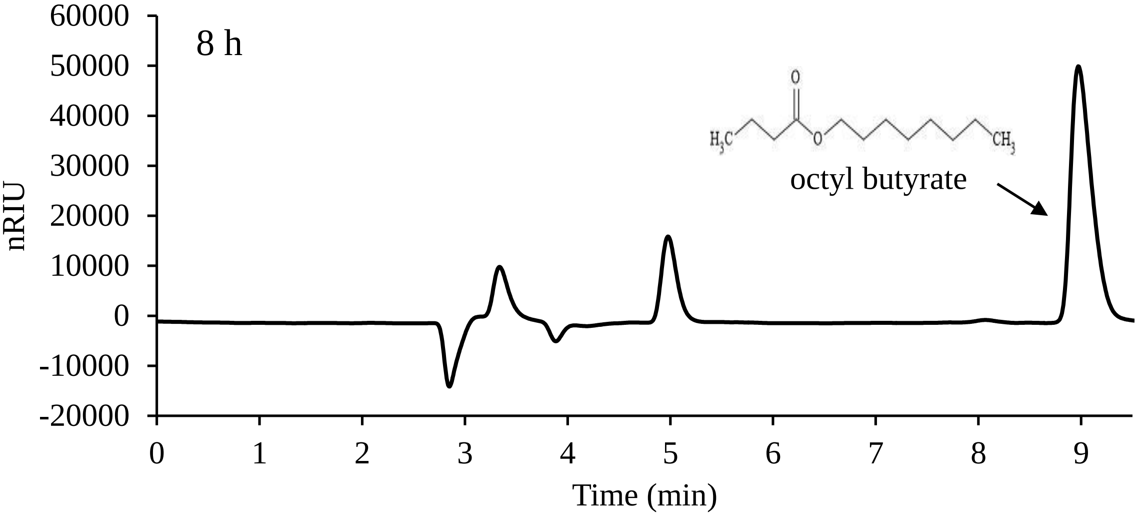

Fig. S1. HPLC analysis of decyl butyrate (E)

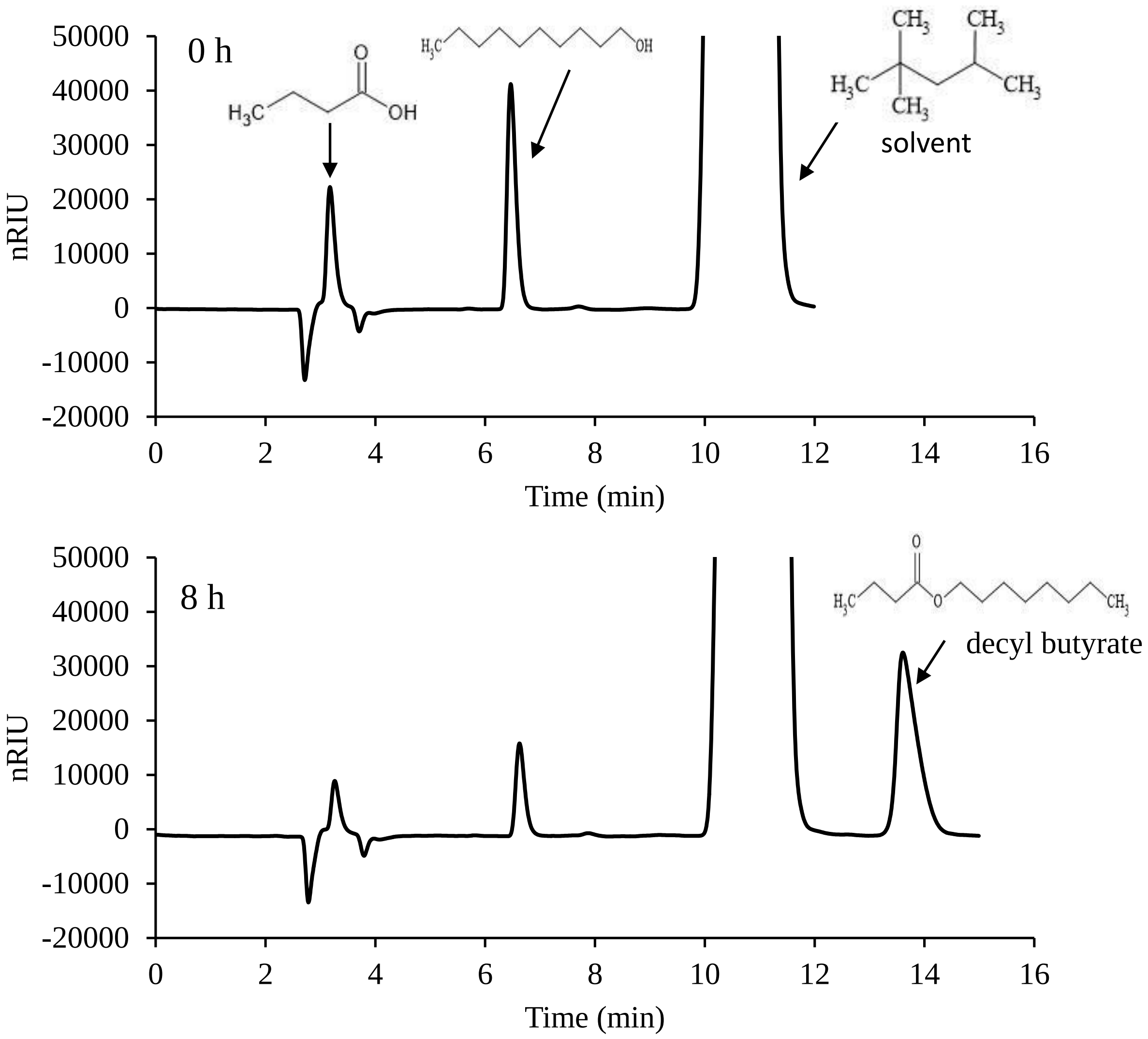

Fig. S1. HPLC analysis of one-pot reaction using butanol, hexanol, and octanol (F)
a, butyric acid; b, butanol; c, hexanol; d, octanol; b’, butyl butyrate; c’, hexyl butyrate; d’, octyl butyrate

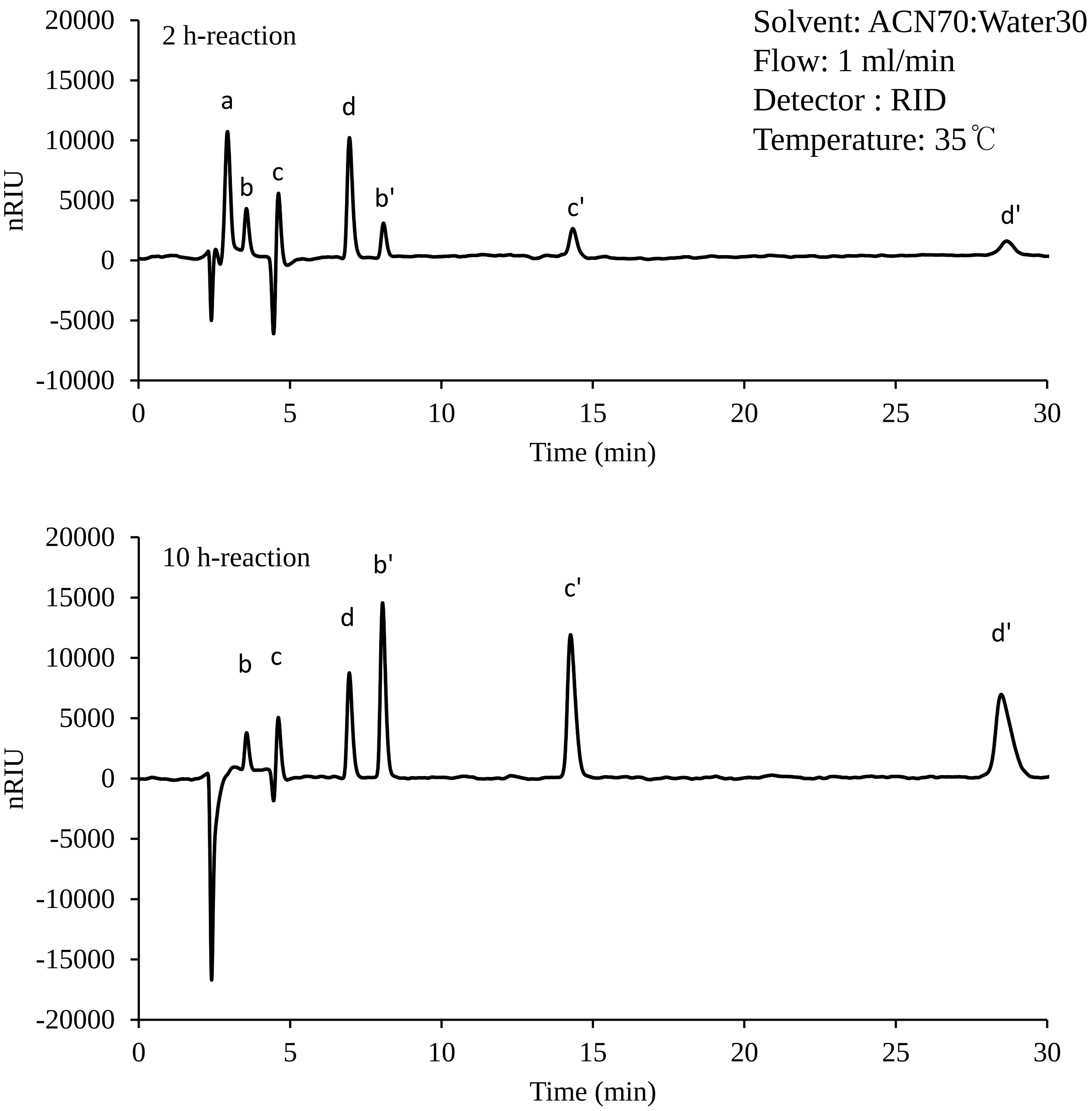

Fig. S2. Standard molarity and equation for each products

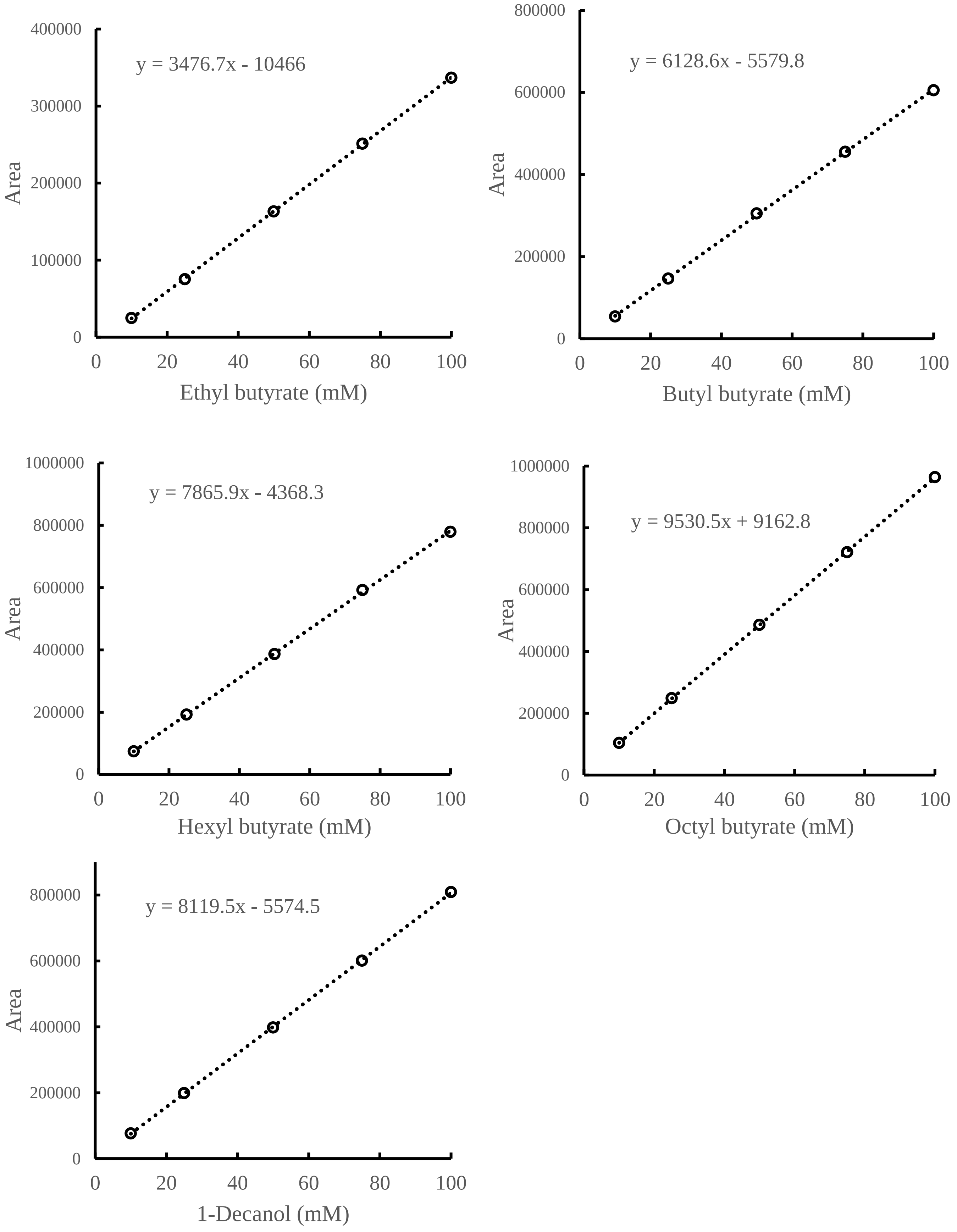

Fig. S3. Alkyl butyrate synthesis and molecular docking results using FoC.

A, Alkyl butyrate synthesis using FoC [18]. B–E, molecular docking result of butanol (B), petanol (C), and heptanol(D).

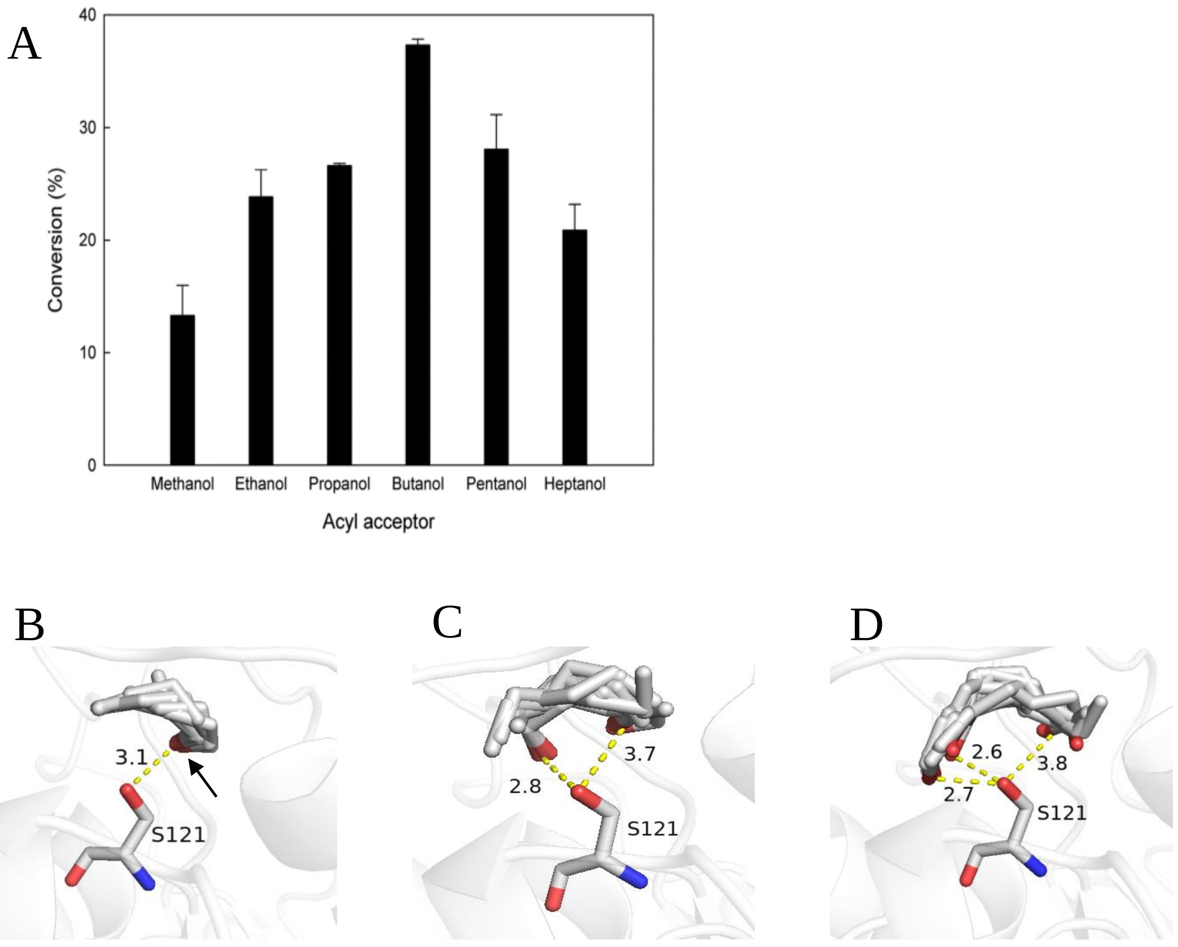

Supplement: Supplementary file 1 [file jmb-33-2-268-supple.pdf]
